# Large language model-assisted research question development in public health: a case study in the Special Supplemental Nutrition Program for Women, Infants, and Children

**DOI:** 10.1017/S1368980026101876

**Published:** 2026-02-02

**Authors:** Qi Zhang, Bidusha Neupane, Priyanka Patel, Futun N. Alkhalifah, Yi He, Leslie Hodges

**Affiliations:** 1 Department of Health Behavior, Policy & Management, Joint School of Public Health, Macon & Joan Brock Virginia Health Sciences at Old Dominion University, Norfolk, VA, USA; 2 School of Computing, Data Sciences & Physics, William & Mary, Williamsburg, VA, USA; 3 U.S. Department of Agriculture, Economic Research Service, Kansas City, MO, USA

**Keywords:** Women, Infants, and Children, Large language model, Artificial intelligence, Nutrition

## Abstract

**Objective:**

To assess the feasibility of using large language models (LLM) to develop research questions about changes to the Special Supplemental Nutrition Program for Women, Infants, and Children (WIC) food packages.

**Design:**

We conducted a controlled experiment using ChatGPT-4 and its plugin, MixerBox Scholarly, to generate research questions based on a section of the U.S. Department of Agriculture (USDA) summary of the final public comments on the WIC revision. Five questions weekly for 3 weeks were generated using LLM under two conditions: fed with or without relevant literature. The experiment generated ninety questions, which were evaluated using the Feasibility, Innovation, Novelty, Ethics and Relevance criteria. *t* tests and multivariate regression examined the difference by feeding status, artificial intelligence model, evaluator and criterion.

**Setting:**

The United States.

**Participants:**

Six WIC expert evaluators from academia, government, industry and non-profit sectors.

**Results:**

Five themes were identified: administrative barriers, nutrition outcomes, participant preferences, economics and other topics. Feeding and non-feeding groups had no significant differences (Coeff. = 0·03, *P* = 0·52). MixerBox-generated questions received significantly lower scores than ChatGPT (Coeff. = –0·11, *P* = 0·02). Ethics scores were significantly higher than feasibility scores (Coeff. = 0·65, *P* < 0·001). Significant differences were found between the evaluators (*P* < 0·001).

**Conclusions:**

The LLM applications can assist in developing research questions with acceptable qualities related to the WIC food package revisions. Future research is needed to compare the development of research questions between LLM and human researchers.

Recent advances in large language models (LLM) have led to increasing artificial intelligence (AI) applications in public health^(1,2)^. Compared with traditional language models, such as topic modelling, opinion mining and document summarisation, LLM have a deeper understanding of natural language contexts, enabling them to capture nuanced meanings, infer implied information and handle complex syntactic and semantic structures conveyed through multiple viewpoints^(3,4)^. This allows them to summarise, paraphrase, analyse and interpret large amounts of text data, such as in social media posts, news articles and public comments.

In public health, LLM have been employed in areas such as health communication analysis, epidemiological modelling, misinformation detection related to health issues and public sentiment analysis concerning public health policies^(5–8)^. Despite the considerable number of LLM applications in public health, no research has been conducted on the use of LLM to assist in the development of research questions in public health, which is a critical task in research. Therefore, the logical first step is to assess the feasibility of using LLM to develop research questions with acceptable qualities. In principle, LLM can help overcome the limitations of traditional human-based question development. First, the human-based approach can be time-consuming and costly due to the need to search for and analyse a large amount of applicable information. For example, literature reviews involve labour-intensive manual coding and thematic analysis to extract insights from an extensive collection of existing knowledge^(9,10)^. LLM can develop questions almost immediately at a low cost. However, developing a significant research question may require spontaneous creativity, which can depend on random factors, such as inspiration and previous experience, and not be amenable to being standardised with given fixed conditions (e.g. a limited time or a fixed venue). In contrast, LLM develop questions solely based on the model specifications. Therefore, it is crucial to explore whether LLM can assist human experts in overcoming these limitations and efficiently developing meaningful research questions.

In this study, we applied LLM to develop research questions related to the Special Supplemental Nutrition Program for Women, Infants, and Children (WIC), the third-largest nutrition assistance program in the United States, which provides nutritious food benefits to more than 6 million low-income eligible women, infants and children under age 5^(11)^. On 21 November 2022, the US Department of Agriculture (USDA) published a proposed rule to update the WIC food package based on a scientific review of the current food packages and updated nutritional science^(12)^. These changes aimed to provide broader choices of foods aligned with the latest nutritional guidelines and more flexibility for the WIC state agencies’ package prescriptions so they could accommodate participants’ special needs and preferences^(13)^. During the public comment period until 21 February 2023, the USDA received 17 731 comments, including 1795 unique comments (993 substantive and 802 categorised comments), 15 863 comments from 16 letter campaigns and 73 duplicates or irrelevant comments. In April 2023, the USDA published a narrative summary of these comments in a document of 159 pages^(14)^. The final rule of the WIC food package revision was published on 18 April 2024, and was effective on 17 June 2024^(15)^.

Even the summary of the large volume of public comments poses a significant challenge for researchers to process and synthesise^(16)^. Large volumes of information can lead to cognitive overload^(17)^, where important details or nuanced opinion stances may be overlooked or inadequately considered. LLM excel at processing large amounts of text efficiently. They can analyse extensive datasets, identifying key themes, sentiments and patterns, thereby aiding in generating comprehensive research questions that represent the public’s views^(18,19)^. LLM have been utilised to analyse massive amounts of social media data, forums and online discussions to understand social movements, cultural trends and public sentiment on various societal topics^(1,20)^.

The recent advances in AI motivate us to examine whether LLM could be used to quickly synthesise large volumes of information, such as public comments on a proposed rule, and generate research questions that will be at least acceptable to the WIC experts. In essence, the goal is to assess the feasibility of using LLM to generate WIC-related research questions and to use human experts to evaluate their qualities against established criteria. If successful, future research can compare the development of research questions between LLM and human researchers. Such an approach could potentially reduce the burden on researchers to sift through thousands of comments manually in future research.

## Methods

### Knowledge base and artificial intelligence model selection

As a case study, we selected the text from section 4.2.2 of the USDA’s final summary as the knowledge base for the AI models. The section summarised public comments regarding the proposal to allow participants to receive a cash value voucher (CVV) or cash value benefit (CVB), a dollar amount that can be used to purchase fruits and vegetables, in place of the juice included in their benefits (i.e. $3 for fruits and vegetables in place of 64 ounces of juice) due to high sugar content and lack of fibre in the juice.

According to the summary, approximately 4249 commenters expressed their opinions regarding this matter. Among them, 1945 commenters favoured allowing CVV as a substitute for the juice benefits, four opposed the proposed change and about 2300 had mixed opinions about the proposed change.

Generative Pretrained Transformer (GPT) models^(1,21)^ are the primary LLM used in the field of natural language understanding and summarisation. In this study, we leveraged GPT-4, a state-of-the-art GPT model with a user-friendly ChatGPT web interface hosted by OpenAI. We refer to this model as *ChatGPT-4*. A unique trait of ChatGPT-4 is that it allows redevelopment after domain-specific training, i.e. it specialises in a specific subject field. Each of such redeveloped GPT models is called a GPT plugin. In this study, we surveyed multiple plugin models. We decided to select MixerBox Scholarly AI (*Mixerbox* for short), which has been retrained with millions of academic documents, papers, research reports, theses and other scholarly resources spanning various domains, including science, engineering, humanities, social sciences, medicine, law and more. A comparison between ChatGPT-4 and Mixerbox would allow for analysing the difference between the responses from either a general or a more academia-specialised AI model.

### Question development experiment

Since ChatGPT-4 is not open-coded, researchers cannot access the algorithm to understand how these questions are developed. Therefore, we created different conditions to compare the results. First, we employed two different approaches to utilising the AI models: ‘feeding’ and ‘non-feeding.’ Under ‘feeding conditions,’ we trained AI models to understand the research questions from the existing literature and then develop research questions based on the given summary. Under ‘non-feeding conditions,’ we directly asked the AI models to generate research questions after reading the summary.

Under the feeding condition approach, it was necessary to train AI models (ChatGPT-4 and Mixerbox) in the WIC context using relevant peer-reviewed research articles. To select the articles, three researchers independently conducted a comprehensive literature review on the related topics, including CVV/CVB, juice, fruits and vegetables in the WIC program. The search was based on three databases: PubMed, Medline and CINAHL between January and February 2024 and utilised specific combinations of keywords and controlled vocabulary terms, including (‘Women, Infant, and Children’ OR WIC) AND (juice OR beverage) OR (fruit* OR vegetable* OR ‘fresh produce’) OR (CVV OR CVB OR ‘cash value benefit’ OR voucher) AND (consumption OR redemption OR substitution). We included only peer-reviewed publications that focused on the WIC program, specifically addressing CVV/CVB substitution, fruit/vegetable intake or juice-related benefits; quantitative, qualitative or mixed-methods study designs and publications in the English language between 2010 and 2023. Studies solely focused on juice consumption, non-peer-reviewed or published in a language other than English were excluded. Following the PRISMA guidelines^(22)^, we identified an initial total of 1508 articles. After removing 758 duplicates, 749 articles were further screened for eligibility based on inclusion and exclusion criteria. Following the full-text review, a total of twenty-one articles were selected for this experiment (selected articles provided in online supplementary material, Supplemental Table 1). The reviewers extracted key components from each article, including the author’s name, publication year, study population, study design and key findings, to verify the selection of articles. Then, each selected article was used to feed and train AI models. The experiment was conducted in May 2024, and the Institutional Review Board at the first author’s institution approved it as an exempt study. All three researchers provided the knowledge base (section 4.2.2 of the USDA summary of public comment on the food package revisions) to the AI models. Then, the researchers independently obtained research questions from the AI models with the following arrangements:

#### Non-feeding approach (i.e. default approach)

One researcher asked the two AI models to develop five research questions weekly for 3 weeks to assess the robustness of the questions and the assumption that the LLM may evolve. Therefore, thirty questions were developed (five questions per week × 2 models (ChatGPT-4 *v*. MixerBox) × 3 weeks).

#### Feeding approach

The other two researchers provided the AI models with the articles identified in the literature review and asked the AI models to summarise the research questions for each article. Then, the researchers asked the AI models to use the USDA summary of public comments to develop five questions per week for 3 weeks. Since the AI models may provide answers based on the user’s profiles and chat history, we employed two researchers to feed the literature to balance the potential user bias from the AI models.

Altogether, the three researchers developed ninety questions (five questions per week × 2 models (ChatGPT-4 *v*. MixerBox) × 3 weeks × 3 researchers). The questions were reviewed using the Delphi technique^(23)^. The research panel, comprising five members – a WIC research expert, an expert in LLM and three other researchers – conducted repeated group discussions to summarise the results into five themes: administrative barriers, nutrition outcomes, participant preferences, economics and general themes. Initially, three researchers independently extracted the questions into separate Excel sheets. Each researcher then categorised the questions into themes independently. During a consensus meeting with a WIC expert, the themes were reviewed and refined. In cases of disagreement, all researchers voted on the appropriate theme, and the theme receiving the majority of votes was selected.

### Evaluation

We asked six evaluators with extensive experience in WIC research to use the Feasibility, Innovation, Novelty, Ethics and Relevance (FINER) criteria to evaluate the quality of the research questions. The FINER criteria were developed by Hulley and colleagues^(24)^ to evaluate clinical research questions and have been widely used in medicine and public health^(25–27)^. We also asked the evaluators to assess the overall quality of the questions. The six evaluators were members of the Healthy Eating Research/Nutrition and Obesity Policy and Research Network (NOPREN) WIC learning collaborative, a national network of WIC researchers, policymakers and practitioners. They represented diverse professional backgrounds, contributing to the broad perspective of this experiment. Of the six, two evaluators were from government agencies, two were from academia and one was from industry and the last was from a non-profit organisation. One of the evaluators had 5 years or less of WIC-related research experience. All other evaluators had more than 5 years of experience in WIC research.

Each evaluator graded three sets of questions to help us identify differences in the quality of research questions based upon:

#### Feeding v. non-feeding approach

The first set consisted of ten pairs of questions randomly selected from ninety questions based on feeding *v*. non-feeding (a total of twenty questions). The randomisation procedure was as follows: Choose randomly ten questions from the feeding group, then randomly select a question from a non-feeding group that matches the same week, theme and AI model.

#### ChatGPT-4 *v.* MixerBox

The second set was ten pairs of questions randomly selected from ninety questions based on the AI models (ChatGPT-4 *v*. MixerBox) (a total of twenty questions). Each pair had the same week, theme and feeding status.

The evaluators scored the questions in sets 1 and 2 on each of the FINER criteria as well as overall quality using a Likert Scale from 1 (very low quality) to 5 (very high quality).

### Data analyses

Descriptive statistics were estimated, including mean, sd, minimum, and maximum scores. A *t* test was performed to determine whether there was a statistically significant difference in scores by feeding status and by AI model. Given the limited sample size for examining the relationship between the quality score and the underlying factors, including feeding status, AI model, evaluator and criterion, we pooled all the scores and conducted a multivariate linear regression. In the randomisation and evaluation process, three questions were repeatedly drawn twice and scored by all six evaluators, which generated a total of 216 scores (3 questions × 2 times × 6 criteria × 6 evaluators). To evaluate whether the repeated evaluation was robust and to control for the clustering effect across time, a generalised estimating equation regression model was applied^(28)^. The score assigned to the questions was the outcome variable, and time (first time or second time), evaluator (evaluator 1–6) and score criterion (FINER and overall) were independent variables. Feasibility was selected as the reference category for the score criterion, as feasibility is the foundation for any meaningful research question^(29)^. The analyses were conducted using R Studio^(30)^.

## Results

Since each of the six evaluators had forty research questions to grade based on six criteria, the total number of scores is 1440 (40 × 6 × 6) (see the distribution of the graded question criteria in Appendix 1). Table 1 presents the ten questions with the highest or lowest mean scores across the five domains (general themes, nutrition outcomes, administrative barriers, participant preferences and economics). In general, the top-rated questions were more specific than the lowest-rated ones. For example, regarding administrative barriers, the top-rated question was ‘*What are the administrative and technological challenges faced by WIC state agencies in implementing multiple CVV substitution options within their MIS systems*?’ while the lowest-rated question was, ‘*What are the administrative and operational implications of multiple CVV substitution options on the WIC program’s Management Information Systems (MIS)*?’ In the top-rated question, the AI clearly understood that the WIC state agencies managed the MIS system and specifically asked about the challenges in implementing multiple CVV substitutions. However, in the lowest-rated question, the question was much vaguer, and the implication was unclear.

**Table 1.** List of research questions with highest or lowest mean scores across themes

Theme	Rating	Mean	sd	Questions
General	Highest	4·33	0·81	Q1a. What are the broader social and equity implications of the CVV substitution policy, especially for low-income families, in accessing nutritious foods and managing dietary conditions such as diabetes?
	Lowest	3·00	1·09	Q1b. How do perceptions and support vary among different stakeholders (e.g. WIC state agencies, healthcare professionals, advocacy groups) regarding the substitution of juice with CVV?
Nutrition outcome	Highest	4·33	0·51	Q2a. What impact does the amount of CVV have on the dietary habits of WIC participants, particularly in terms of accessing and consuming a wider variety of fruits and vegetables?
	Lowest	2·66	1·21	Q2b. What are the equity implications of allowing juice to be swapped for CVV, particularly in regard to vitamin C consumption among WIC participants?
Administrative barriers	Highest	4·16	0·75	Q3a. What are the administrative and technological challenges faced by WIC state agencies in implementing multiple CVB substitution options within their MIS systems?
	Lowest	3·16	0·98	Q3b. What are the administrative and operational implications of multiple CVB substitution options on the WIC program’s MIS?
Participants’ preference	Highest	4·00	1·09	Q4a. How does the substitution of juice with CVV influence consumer choice and nutrition in populations with restricted access to fresh produce, such as those in food deserts or experiencing homelessness?
	Lowest	2·50	1·22	Q4b. How do participant preferences and choices influence the effectiveness of allowing CVV as a substitute for juice in the WIC program?
Economics	Highest	3·83	1·32	Q5a. How do market value discrepancies and geographic variations in the cost of living affect the adequacy of the CVV amount as a substitute for juice in meeting the nutritional needs of WIC participants?
	Lowest	2·33	1·21	Q5b. What are the economic implications of the proposed increase in CVV amount to align with the nutritional and cost value of juice for both participants and the program?

WIC, Women, Infant, and Children; CVV, cash value voucher; CVB, cash value benefits; MIS, management information systems.

Table 2 presents the *t* test results of the quality scores by feeding status (feeding *v*. non-feeding questions), which had no statistically significant differences across the FINER criteria or overall quality. For example, the mean overall score for the feeding group was 3·41 (sd = 1·05), whereas for the non-feeding group, it was 3·54 (sd = 1·07) (*P* = 0·36). Table 2 presents the *t* test results of the quality scores by AI model (GPT-4 *v*. MixerBox questions). Similarly, there was no significant difference across all six criteria. For example, the mean overall score for GPT-4 was 3·55 (sd = 1·01) compared with 3·40 (sd = 1·11) for MixerBox (*P* = 0·25).

**Table 2.** Summary of research question scores by feeding conditions and AI models (*n* 1440)

Traits	Feeding Mean	Feeding sd	Non-Feeding Mean	Non-Feeding sd	Difference of Means	*P* value*
Feasibility	3·41	0·83	3·47	0·80	−0·05	0·58
Interest	3·49	1·14	3·54	1·14	−0·05	0·73
Novelty	3·61	1·08	3·56	1·11	0·05	0·72
Ethics	4·11	0·85	4·09	0·80	0·02	0·72
Relevance	3·57	1·09	3·60	1·13	−0·03	0·86
Overall	3·41	1·05	3·54	1·07	−0·13	0·36
	GPT4 Mean	GPT4 sd	Mixer-Box Mean	Mixer-Box sd		
Feasibility	3·49	0·77	3·40	0·86	0·09	0·38
Interest	3·61	1·10	3·41	1·17	0·20	0·17
Novelty	3·62	1·03	3·55	1·15	0·07	0·63
Ethics	4·09	0·78	4·11	0·87	−0·02	0·81
Relevance	3·67	1·04	3·50	1·17	0·17	0·22
Overall	3·55	1·01	3·40	1·11	0·15	0·25

AI, artificial intelligence; *t test.

Table 3 presents mean scores and sd of scores assigned by each evaluator to all six criteria (FINER + O). The result shows that Evaluator 1 consistently assigned lower scores compared to all other evaluators. For instance, the mean score for relevance was 2·33 (sd = 1·07), whereas the mean score of other evaluators for the same criteria ranged from 3·50 to 4·20 (sds ranged from 0·65 to 1·32). This shows that Evaluator 1 was more stringent in interpreting the criterion than other evaluators.

**Table 3.** Mean scores by evaluator and criteria (*n* 1440)

Criteria												
Evaluator	Feasibility		Interest		Novelty		Ethics		Relevance		Overall	
	M	sd	M	sd	M	sd	M	sd	M	sd	M	sd
1	3·10	0·67	2·15	1·17	2·30	1·09	3·68	0·57	2·33	1·07	2·30	1·07
2	3·45	0·75	3·65	0·66	3·43	0·59	4·73	0·55	3·68	0·66	3·75	0·44
3	3·38	0·81	3·68	0·73	3·68	0·80	3·63	0·77	3·50	0·91	3·45	0·88
4	3·40	0·74	3·75	0·84	4·40	0·71	4·43	0·55	4·20	0·65	3·75	0·74
5	3·83	1·03	3·88	1·30	3·93	0·92	4·93	0·27	3·95	1·32	3·80	1·34
6	3·53	0·75	4·00	0·93	3·83	1·13	3·25	0·49	3·88	0·88	3·83	0·84

M, mean.

Table 4 presents the regression results of the scores on feeding status, model, evaluator and criteria. Feeding status, i.e. whether the model was given additional literature before generating the questions, was not significantly related to the quality scores of the research questions (*β* = 0·03, *P* = 0·52). However, the difference in model type was statistically significant with questions generated by the MixerBox model being associated with lower average scores compared with questions generated by ChatGPT-4 (*β* = −0·11, *P* = 0·02). Scores given by all other evaluators were significantly higher compared with scores given by the reference evaluator (Evaluator 1), who is a senior WIC researcher (*P* < 0·001). Finally, scores based on the ethics criteria were significantly higher compared with scores based on feasibility (*β* = 0·65, *P* < 0·001). Scores based on other criteria, such as interest, novelty, relevance and overall quality, did not show significant differences in this model.

**Table 4.** Regression results of quality scores on conditions (*n* 1440)

Independent variables	Estimate (*β*)	se	*P* value
Feeding status			
Feeding *(Ref)*			
Non-feeding	0·03	0·04	0·52
GPT Model			
GPT-4 (Ref)			
Mixerbox	–0·11	0·04	0·02
Evaluators			
Evaluator1 *(Ref)*			
Evaluator 2	1·14	0·08	< 0·001
Evaluator 3	0·91	0·08	< 0·001
Evaluator 4	1·35	0·08	< 0·001
Evaluator 5	1·41	0·08	< 0·001
Evaluator 6	1·08	0·08	< 0·001
Score criteria			
Feasibility *(Ref)*			
Interest	0·07	0·08	0·39
Novelty	0·14	0·08	0·07
Ethics	0·65	0·08	< 0·001
Relevance	0·14	0·08	0·08
Overall	0·03	0·08	0·68

se, standard error.

Table 5 presents the results of generalised estimating equation analyses of the repeated measures of the 216 scores. There was no significant difference across evaluation time (*β* = –0·05, *P* = 0·52). Similar to the results in Table 3, the evaluators and the ethics criterion were significantly related to the scores. The evaluator effect was highly significant across all the evaluators compared with the reference evaluator (Evaluator 1) (*P* < 0·001). All the evaluators provided significantly higher scores than Evaluator 1. With feasibility as a reference criterion, the ‘ethics’ criterion showed a significant difference, yielding a higher score (*β* = 0·44, *P* = 0·04).

**Table 5.** Generalized Estimate Equation (GEE) for repeated evaluation (*n* 216)

Independent variables	Estimate(*β*)	se	*P* value
Time			
First time *(Ref)*			
Second time	−0·05	0·07	0·44
Evaluators			
Evaluator 1 *(Ref)*			
Evaluator 2	1·17	0·17	< 0·001
Evaluator 3	1·14	0·16	< 0·001
Evaluator 4	1·42	0·21	< 0·001
Evaluator 5	1·97	0·16	< 0·001
Evaluator 6	1·19	0·22	< 0·001
Score criteria			
Feasibility *(Ref)*			
Interest	0·08	0·20	0·68
Novelty	−0·14	0·22	0·52
Ethics	0·44	0·22	0·04
Relevance	0·11	0·21	0·58
Overall	0·06	0·21	0·78

## Discussion

Although this is a pilot study, the findings indicate that AI has the potential to produce research questions related to the WIC program that experienced scholars in the field consider to be of sufficient quality and relevance. Based on the five top-rated questions in Table 1, researchers may benefit from this exercise by incorporating these questions into their future WIC research projects. The general theme question (Q1a) asks about the social and equity implications of the CVV substitution policies related to dietary conditions among low-income populations. Given the increasing trend in gestational diabetes in the USA and the particular vulnerability of low-income mothers to this health condition^(31)^, it is important to examine how the reduction of juice intake with CVV substitution can benefit these WIC mothers with gestational diabetes. Moreover, with CVV substitution, WIC mothers can benefit from a wider choice of fruits and vegetables. However, its impact is worth evaluating, so Q2a under nutritional outcomes highlights this knowledge gap.

Q3a focuses more on the implementation of the substitution policy. Researchers may adopt a mixed-method approach to answer that question. Q4a poses a serious question regarding the potential limitation of this CVV substitution, as some WIC participants may not have sufficient access to fruits and vegetables. Therefore, they may not fully benefit from the new policy. Thus, a series of studies can be launched to evaluate the behaviours of WIC participants after the CVV substitution policy is implemented, especially among those with limited fruits and vegetables access, such as in food deserts. Q5a is less significant but still meaningful, as varying costs of living and high inflation may limit participants’ motivation to substitute juice with CVV, given the cost-effectiveness of the juice^(32)^. Therefore, these five questions all have the potential to develop research lines to fully understand the implementation and impact of the CVV substitution policies.

The research questions generated by the AI models indicated areas where future research on CVV substitution could improve the current understanding of the program’s effectiveness. For instance, the research question regarding the broader social and equity implications of the CVV substitution policy, particularly among low-income families and individuals with diabetes, is relevant to WIC’s objectives of reducing dietary disparities and promoting health. This highlights the need for future research to assess how changes in WIC policies may impact vulnerable populations, as the findings can be crucial in addressing shifts in the amount of CVV and in designing policies to meet the needs of vulnerable groups, such as women or children with diabetes.

The evaluators agree more consistently on the ethical soundness of the generated questions compared with their feasibility, novelty or relevance to the research community. We attribute this observation to recent advances in AI ethics research^(33)^, which focus on aligning AI systems with human values, enabling them to identify and adhere to ethical norms and societal consensus. A prominent approach to achieving AI alignment involves incorporating fairness, accountability and transparency principles into training through reinforcement learning from human feedback^(34)^. Reinforcement learning from human feedback ensures that AI systems prioritise ethical considerations and minimise biases, adapting their generated content into sensitive WIC research settings, such as focusing on equity, access and the needs of vulnerable populations. However, we also note that the full integration of AI into WIC contexts would require domain-specific data to capture the complexities of public health research and policy analysis more effectively. For example, enabling the models to understand the socio-economic implications of WIC policies could enhance their ability to generate questions that are both ethically sound and practically relevant^(35)^. These advances in AI ethics research further explore another critical capability of AI: its potential to stimulate curiosity.

In addition to having the potential to prompt members of the WIC community to utilise AI for research on WIC, the findings from this study also hold value for AI researchers. In particular, generating meaningful and insightful research questions inherently requires curiosity, an attribute traditionally considered a hallmark of human cognition^(36)^. The results suggest that these models possess a rudimentary form of curiosity, or at least the ability to simulate it in a manner that produces outcomes similar to human inquiry^(37)^. Moreover, this study used the summary comments generated by the USDA. In future work, LLM may be used to create questions based on the original comments, as summarising the large volume of original comments can be time-consuming, introduce bias and result in random errors.

Although this is the first case study to utilise LLM in developing WIC-related research questions, we acknowledge several limitations. First, since we needed to test the feeding *v*. no feeding condition, we focused on one section of the summary so that we could conduct a literature review related to that particular section. However, this limitation restricts the broader scope of LLM in generating research questions based on the complete summary. Second, this study cannot specify the number and the quality of research questions generated by human researchers. Therefore, we could not compare the question generation between LLM and a human researcher, leaving the question of whether LLM could effectively replace or augment human researchers in generating research questions unsettled. Moreover, the ethical findings of the AI-generated questions are only applicable to the specific topic studied and may not be extended to other, more complex or controversial issues. Finally, we did not test other GPT plugins that were available. As a pioneering study, we presented preliminary results to demonstrate the feasibility of using LLM to generate research questions.

Beyond the technical findings of this study, the use of AI to generate research questions raises important ethical, moral and societal considerations. The formulation of research questions has traditionally been viewed as a core intellectual responsibility of researchers, reflecting their domain expertise, creativity and ability to integrate diverse sources of knowledge. Delegating this task to AI systems such as LLM inevitably prompts broader questions about the role of human judgement in research and the extent to which AI might eventually replace or reshape this role^(38,39)^. While parallels may be drawn to asking AI to draft grants or manuscripts, we emphasise that our findings cannot speak to the question of whether AI can replace human researchers in this or other scholarly activities, as our study did not include a direct comparison between AI-generated and human-generated research questions. This study has considered one way that AI could be used as a supplementary tool to enhance efficiency and contribute to idea generation, but whose outputs still require human oversight for contextual relevance, originality and alignment with disciplinary standards^(40)^. Future research may be able to examine these comparisons and engage in deeper dialogue on the appropriate and ethical integration of AI into the research process.

### Conclusions

In conclusion, LLM may be a valuable tool for analysing WIC-related text data to generate acceptable research questions for WIC. They potentially can be used for broader health-related research. Our results demonstrate sufficient merit for future comparisons between AI approaches and human researchers in developing research questions, ultimately leading to an assessment of whether AI can replace or augment human researchers in this area. This study suggests that using a general-purpose GPT model may be sufficient for producing thematically relevant questions without the need for additional literature feeding or specialised plugins. However, more research is warranted as these conclusions cannot be generalised to other topics. As AI continues to advance, research studies like ours can inform the understanding of how to combine the efficiency of LLM with human expertise to generate policy-relevant, discipline-appropriate research questions.

## Supporting information

Zhang et al. supplementary material Zhang et al. supplementary material

## Supplementary material

For supplementary material accompanying this paper visit https://doi.org/10.1017/S1368980026101876.

## Appendix 1. Distributions of graded research question criteria across conditions (*n* 1440)

**Table:**

Independent variables	Number (*n*)
Feeding status	
Feeding	720
Non-feeding	720
GPT model	
GPT-4	720
Mixerbox	720
Evaluators	
Evaluator 1	240
Evaluator 2	240
Evaluator 3	240
Evaluator 4	240
Evaluator 5	240
Evaluator 6	240
Score criteria	
Feasibility	240
Interest	240
Novelty	240
Ethics	240
Relevance	240
Overall	240
