# Supplementary material for: Large language model-assisted research question development in public health: a case study in the Special Supplemental Nutrition Program for Women, Infants, and Children
Source: Public Health Nutr. 2026 Feb 2;29(1):e32. doi: 10.1017/S1368980026101876 (PMC12917417; doi:10.1017/S1368980026101876)
Supplement: Zhang et al. supplementary material [file S1368980026101876sup001.docx]

| Ishdorj and Capps^(9)^ | 2017 | Texas | Juice | Consumption & Redemption | Consumption of 100.0% juice was higher than that of sugary and artificially sweetened beverages. However, total juice purchases decreased. |
| --- | --- | --- | --- | --- | --- |
| Zhang et al.^(10)^ | 2017 | Virginia | F&V | Redemption | Minority participants redeemed higher-priced brands of infant fruits and vegetables. |
| Guthrie et al.^(11)^ | 2018 | National | Vegetables | Consumption | WIC children are more likely to consume infant vegetables than non-WIC. |
|  |  |  | Juice | Consumption | WIC children are more likely to drink juice than non-WIC. |
| Ng et al.^(12)^ | 2018 | National | F&V | Redemption | Increased purchases of fruits and vegetables. |
|  |  |  | Juice | Redemption | Decreased purchases of sugar-sweetened drinks, including juice. |
| Vercammen et al.^(13)^ | 2018 | National | F&V | Consumption | No difference in consumption of whole fruit and total vegetables between WIC participants and non-participants. |
|  |  |  | Juice | Consumption | WIC participants consumed significantly more 100.0% fruit juice than nonparticipants, exceeding the age-specific American Academy of Pediatrics maximum intake for juice. |
| Charvet et al.^(14)^ | 2019 | Florida | Juice | Consumption | WIC children consume more than twice the recommended amount of 100.0% fruit juice per day. |
| Hamner et al.^(15)^ | 2019 | National | Fruits | Consumption | WIC participants consume more fruit than non-participants. |
|  |  |  | Juice | Consumption | WIC participants consume more 100.0% juice compared to non-participants. |
| Zimmer et al.^(16)^ | 2019 | National | F&V | Consumption | Starchy vegetable consumption decreased, but no changes in fruit consumption. |
| Guthrie et al.^(17)^ | 2020 | National | F&V | Consumption | WIC children consumed more fruits and vegetables in 2016 compared to 2008. |
|  |  |  | Juice | Consumption | In 2016, WIC children consumed more juice than non-WIC children. |
| Zhang et al.^(18)^ | 2021 | West Virginia & Kansas | F&V | Redemption | App users showed higher F&V redemption compared to non-app users. |
|  |  |  | Juice | Redemption | Increased redemption for frozen juice (28.0% West Virginia; 48.0% Kansas) and shelf-stable juice (13.0% West Virginia; 20.0% Kansas). |
| Gago et al.^(19)^ | 2022 | Massachusetts | F&V | Consumption & Redemption | Increasing WIC allotments for CVB led to increased purchase and consumption of fruits and vegetables. |
| Halverson and Karpyn^(20)^ | 2022 | Delaware | F&V | Consumption & Redemption | CVB increase promoted the purchase and consumption of fruits and vegetables during the COVID-19 pandemic. |
| Whaley et al.^(21)^ | 2023 | California | F&V | Consumption & Redemption | Increased CVB correlated with greater redemption of fruits and vegetables. WIC children with the lowest baseline intake in 2021 showed increased fruit and vegetable consumption in 2022. |

^*^LLMs, language learning models; F&V, fruit and vegetable; CVV, cash value voucher; WIC, Women, Infants, and Children; CVB, cash value benefits.

**References**

1. Andreyeva T, Luedicke J, Tripp AS, *et al.* (2013) Effects of reduced juice allowances in food packages for the Women, Infants, and Children program. *Pediatrics* **131**, 919–927.
2. Kim LP, Whaley SE, Gradziel PH, *et al.* (2013) Mothers prefer fresh fruits and vegetables over jarred baby fruits and vegetables in the new Special Supplemental Nutrition Program for Women, Infants, and Children food package. *J Nutr Educ Behav* **45**, 723–727.
3. Beck AL, Takayama JI, Halpern-Felsher B, *et al.* (2014) Understanding how Latino parents choose beverages to serve to infants and toddlers. *Matern Child Health J* **18**, 1308–1315.
4. Kong A, Odoms-Young AM, Schiffer LA, *et al.* (2014) The 18-month impact of Special Supplemental Nutrition Program for Women, Infants, and Children food package revisions on diets of recipient families. *Am J Prev Med* **46**, 543–551.
5. Odoms-Young AM, Kong A, Schiffer LA, *et al.* (2014) Evaluating the initial impact of the revised Special Supplemental Nutrition Program for Women, Infants, and Children (WIC) food packages on dietary intake and home food availability in African-American and Hispanic families. *Public Health Nutr* **17**, 83–93.
6. Andreyeva T, Luedicke J (2015) Incentivizing fruit and vegetable purchases among participants in the Special Supplemental Nutrition Program for Women, Infants, and Children. *Public Health Nutr* **18**, 33–41.
7. Morshed AB, Davis SM, Greig EA, *et al.* (2015) Effect of WIC food package changes on dietary intake of preschool children in New Mexico. *Health Behav Policy Rev* **2**, 3–12.
8. Reat AM, Crixell SH, Friedman BJ, *et al.* (2015) Comparison of food intake among infants and toddlers participating in a South Central Texas WIC program reveals some improvements after WIC package changes. *Matern Child Health J* **19**, 1834–1841.
9. Ishdorj A, Capps O (2017) The impact of policy changes on milk and beverage consumption of Texas WIC children. *Agric Resour Econ Rev* **46**, 421–442.
10. Zhang Q, Tang C, McLaughlin PW, *et al.* (2017) Individual and store characteristics associated with brand choices in select food category redemptions among WIC participants in Virginia. *Int J Environ Res* *Public Health* **14**, 364.
11. Guthrie JF, Catellier DJ, Jacquier EF, *et al.* (2018) WIC and non-WIC infants and children differ in usage of some WIC-provided foods. *J Nutr* **148** Suppl. 3, S1547–S56.
12. Ng SW, Hollingsworth BA, Busey EA, *et al.* (2018) Federal nutrition program revisions impact low-income households' food purchases. *Am J Prev Med* **54**, 403–412.
13. Vercammen KA, Moran AJ, Zatz LY, *et al.* (2018)100% juice, fruit, and vegetable intake among children in the Special Supplemental Nutrition Program for Women, Infants, and Children and nonparticipants. *Am J Prev Med* **55**, e11-e18.
14. Charvet A, Huffman FG (2019) Beverage intake and its effect on body weight status among WIC preschool-age children. *J Obes* **2019**, 3032457.
15. Hamner HC, Paolicelli C, Casavale KO, *et al.* (2019) Food and beverage intake from 12 to 23 months by WIC status. *Pediatrics* **143**, e20183448.
16. Zimmer MC, Rubio V, Kintziger KW, *et al.* (2020) Differences in consumption of NASEM priority nutrients and food groups by race/ethnicity among women living in WIC-participating households. *Am J Health Promot* **34**, 791–795.
17. Guthrie JF, Anater AS, Hampton JC, *et al.* (2020) The Special Supplemental Nutrition Program for Women, Infants, and Children is associated with several changes in nutrient intakes and food consumption patterns of participating infants and young children, 2008 compared with 2016. *J Nutr* **150**, 2985–2993.
18. Zhang Q, Zhang J, Park K, *et al.* (2021) App usage associated with full redemption of WIC food benefits: A propensity score approach. *J Nutr Educ Behav* **53**, 779–786.
19. Gago C, Colchamiro R, May K, *et al.* (2022) Caregivers’ perceived impact of WIC’s temporary cash-value benefit (CVB) increases on fruit and vegetable purchasing, consumption, and access in Massachusetts. *Nutrients* **14**, 4947.
20. Halverson MM, Karpyn A (2022) WIC participants’ perceptions of the cash-value benefit increase during the COVID-19 pandemic. *Nutrients* **14**, 3509.
21. Whaley SE, Anderson CE, Tsai MM, *et al.* (2023) Increased WIC benefits for fruits and vegetables increases food security and satisfaction among California households with young children. *J Acad Nutr Diet* **123**, 1440–1448. e1441
